# MANF silencing, immunity induction or autophagy trigger an unusual cell type in metamorphosing *Drosophila* brain

**DOI:** 10.1007/s00018-014-1789-7

**Published:** 2014-12-16

**Authors:** Vassilis Stratoulias, Tapio I. Heino

**Affiliations:** Department of Biosciences, University of Helsinki, P.O. Box 56, Viikinkaari 5, 00014 Helsinki, Finland

**Keywords:** Pupa, Tor, IMD, Atg

## Abstract

Glia are abundant cells in the brain of animals ranging from flies to humans. They perform conserved functions not only in neural development and wiring, but also in brain homeostasis. Here we show that by manipulating gene expression in glia, a previously unidentified cell type appears in the *Drosophila* brain during metamorphosis. More specifically, this cell type appears in three contexts: (1) after the induction of either immunity, or (2) autophagy, or (3) by silencing of neurotrophic factor DmMANF in glial cells. We call these cells MANF immunoreactive Cells (MiCs). MiCs are migratory based on their shape, appearance in brain areas where no cell bodies exist and the nuclear localization of dSTAT. They are labeled with a unique set of molecular markers including the conserved neurotrophic factor DmMANF and the transcription factor Zfh1. They possess the nuclearly localized protein Relish, which is the hallmark of immune response activation. They also express the conserved engulfment receptor Draper, therefore indicating that they are potentially phagocytic. Surprisingly, they do not express any of the common glial and neuronal markers. In addition, ultrastructural studies show that MiCs are extremely rich in lysosomes. Our findings reveal critical molecular and functional components of an unusual cell type in the *Drosophila* brain. We suggest that MiCs resemble macrophages/hemocytes and vertebrate microglia based on their appearance in the brain upon genetically challenged conditions and the expression of molecular markers. Interestingly, macrophages/hemocytes or microglia-like cells have not been reported in the fly nervous system before.

**Electronic supplementary material:**

The online version of this article (doi:10.1007/s00018-014-1789-7) contains supplementary material, which is available to authorized users.

## Introduction

Glia are the most abundant cell type in the mammalian nervous system. They have long been thought to have only supportive roles like insulating and nourishing neurons. However, this concept is rapidly changing as new findings demonstrate that glia play an irreplaceable role in all aspects of the nervous system development and function.

In *Drosophila* three glia classes—surface, cortex and neuropil—glia have been characterized based on their morphology, molecular markers and position [20]. They share many morphological and molecular similarities with their mammalian counterparts [56]. More importantly, they perform similar functions such as providing trophic support for neurons [5], pathfinding and ensheathing of axons. However, there are some pronounced differences between mammalian and *Drosophila* glia. In *Drosophila*, glia number is significantly smaller than in mammals and they are not associated with any type of myelin sheath [12].

Macrophages are highly specialized cells that constitute the cellular immunity of organisms ranging from flies to humans. In mammals, resident macrophages exist in all tissues of the body. They are the first line of defense against injury and infection, responding rapidly to disturbances of tissue homeostasis. The resident macrophages of the mammalian nervous system are called microglia. Surprisingly, and contrary to what their name suggests, microglia do not have a neuro-ectodermal origin. During embryogenesis, microglia derive from macrophages produced in the yolk sac and after their differentiation to microglia in the neural tube, they enter the central nervous system (CNS) (reviewed in [17, 24]). In mammals, where microglia have been studied, they constitute 10 % of the cells in the CNS [31]. Upon infection or damage caused by ischemic and neurodegenerative insults they activate and rapidly move to the damaged area to eliminate infective agents or neuronal debris, while they release neurotrophic factors and pro-inflammatory mediators [23]. However, recent data indicate that the term microglia activation is not an all-or-none process. For instance, the phagocytic function of microglia is impaired in mice with Alzheimer-like pathology [23, 26], as well as in prion diseased brains [44, 53]. Interestingly, upon severe neuroinflammation and/or neurodegeneration, macrophage infiltration can also occur [23]. However, distinction between microglia and infiltrating macrophages is hampered by the overlap of markers expressed in both cell types [23].

Macrophages/hemocytes, or microglia-like cells have not been identified in the fly CNS. During *Drosophila* embryogenesis, apoptotic cell clearance is performed by professional phagocytes, called macrophages [55]. Macrophages, also designated as hemocytes, are found in the hemolymph either as sessile or as freely circulating moieties, being associated with various tissues. Hemocytes display phagocytic and scavenger properties [61]. However, once the nerve cord is ensheated, hemocytes have no longer access in the nervous system [28]. During development sessile glia assume phagocytic role in the *Drosophila* CNS [4, 16, 28, 60]. In adult flies glia act as “semiprofessional” phagocytes engulfing apoptotic neurons [38].

In this study, we describe an unusual cell type in the *Drosophila* pupal brain, that we call MiC. Based on their morphology, unexpected appearance upon certain genetic manipulations and the molecular markers they express, MiCs resemble macrophages/hemocytes and vertebrate microglia.

## Materials and methods

### Fly strains and genetics

Flies were kept and raised under standard conditions at 18, 25 and 26 °C, depending on the genotype (for details see Supplementary Table S1). *w* ^*1118*^ flies were regarded as wild type. We used the following stocks from Bloomington *Drosophila* Stock Centre (BDSC): *He*-*Gal4*, *UAS*-*GFP* (8700), *Hml*-*Gal4*, *UAS*-*GFP* (30140), *UAS*-*PGRC*-*LE* (33054), *UAS*-*PGRC*-*LC* (33917), *UAS*-*Toll* (30900), *UAS*-*Toll* (30901), *UAS*-*DIAP1* (6657), *UAS*-*Tor* ^*TED*^ (7013), *UAS*-*p35* (5072), *UAS*-*p35* (5073), *UAS*-*nGFP* (4775), *UAS*-*mCD8*::*GFP* (5137) and *UAS*-*DmMANF* ^*RNAi*^ (v12834, v12835) and *UAS*-*Neuroglian* ^*RNAi*^ (v107911) [from Vienna *Drosophila* RNAi Centre (VDRC)]. The following stocks are the glial subtype drivers presented and were obtained from the *Drosophila* Genomics Resource Centre (DGRC): NP1243 (12835), NP2222 (112830), NP2276 (112853), NP3233 (113173), NP6293 (105188), NP6520 (105240) and *alrm*-*Gal4* [gift from M. R. Freeman, (HHMI, USA)]. In addition, these stocks were also used: *repo*-*Gal4* (gift from V. J. Auld, UBC, Canada), *elav*-*Gal4* (first chromosome) (BDSC, 458), *elav*-*Gal4* (third chromosome) (BDSC, 8760), *Gcm*-*Gal4* (gift from A. Giangrande, IGBMC, France), *TH*-*Gal4* (gift from S. Birman, Paris Institute of Technology, France), *tub*-*Gal4*, *UAS*-*Dicer*-*2* (second and third chromosome gifts from M. Baumgardt, Linköping University, Sweden), *Nazgul*-*Gal4* (Gift from B. Altenhein, University of Mainz, Germany), *prospero*-*Gal4* (Gift from B. Denholm, University of Cambridge, UK), *UAS*-*grim*, *UAS*-*hid*, -*reaper* and UAS-*hid*, -*reaper*, -*grim* (gifts from Nambu/M. O’Connor, University of Minnesota USA), *UAS*-*Atg1* (GS10797) (gift from T. Neufeld [49], University of Minnesota USA), *UAS*-*Hsap\SNCA.A30P* (BDSC, 8147), *UAS*-*RelD* (gift from S. Cherry, University of Pennsylvania, USA) and *UAS*-*lacZ* (gift from N. Perrimon, HMS, USA).

### MiCs phenotype

MiCs phenotype was reproduced by the following genotypes, both in male and female animals: *UAS*-*DmMANF* ^*RNAi*^; *UAS*-*Dicer*-*2*/*repo*-*Gal4* (at 18 °C), *UAS*-*Dicer*-*2*/*UAS*-DmMANF^RNAi^; *repo*-*Gal4* (at 18 °C), *UAS*-*PGRC*-*LE*; *repo*-*Gal4* (at 26 °C *UAS*-*PGRC*-*LC*; +; *repo*-*Gal4* (at 26 °C), *repo*-*Gal4*/*UAS*-*Atg1* (GS10797) (at 26 °C) and *UAS*-*Tor* ^*TED*^/*repo*-*Gal4* (at 26 °C). Note that *UAS*-*Dicer*-*2* on the second chromosome was giving weaker phenotype compared to *UAS*-*Dicer*-*2* on the third chromosome.

### Time scale

All times indicated represent time of development at 25 °C. *repo*-*Gal4*>*UAS*-*DmMANF* ^*RNAi*^ *UAS*-*Dicer*-*2* flies were raised at 18 °C (as well as other transgenic animals-for details see Supplementary Table S1). Therefore, times shown in Figs. 7, 8, Supplementary Figure S6 and Supplementary Table S1, should be doubled, taking into account that flies have ~½ rate of development at 18 °C, compared to that at 25 °C.

### Immunohistochemistry

All phenotypes and images presented in this study are of late (pharate) pupae unless otherwise indicated. For ensuring that dark pupae were alive on dissection, only first dark pupae from each vial were sacrificed. Brain dissection and immunohistochemistry were performed as described in Wu and Luo [67]. For ovaries, testis and muscle the protocol was changed as follows: 0.1 % PBT and one overnight primary antibody incubation.

In this study, the following antibodies from Developmental Studies Hybridoma Bank were used: Rat anti-Elav (1:20), mouse anti-Engrailed (1:10), mouse anti-Discs large (1:10), mouse anti-Bruchpilot (1:10), mouse anti-Relish-C (1:10), mouse anti-Repo (1:10) and mouse anti-Sim (1:10). In addition, the following antibodies were also used: rabbit anti-DmMANF (1:1,000, [42]), mouse anti-Relish-N (1:100, gift from S. Stöven, Umea University, Sweden), rabbit anti-dSTAT (1:1,000, gift from E. Bach [14], NYULMC, USA), guinea pig anti-Zfh1 (1:500, gift from J. Skeath, Washington University in St. Louis, USA), rabbit anti-Draper [1:500, gift from M. R. Freeman, [16], (HHMI, USA)], rat anti-Draper (1:250, gift from Y. Nakanishi, [39], Kanazawa University, Japan), mouse anti-BrdU (GE Healthcare, 1:400), mouse anti-TH (Diasorin, 1:25), rabbit anti-Phosphohistone3 (Upstate Cell Signaling Solutions, 1:1,000), rabbit anti-Caspase-3 (Cell Signaling Technology, 1:50), rabbit anti-DNP-BSA (ICN ImmunoBiologicals, 1:1,000) and rhodamine phalloidin (1:1,000, Sigma).

Secondary antibodies were obtained from Jackson ImmunoResearch Laboratories: goat anti-mouse and anti-rat F(ab′)_2_ fragments coupled to DyLight 488, 561 or 633 (1:200 for 488 and 633, and 1:400 for 561) and from Molecular Probes: goat anti-rabbit and goat anti-Guinea Pig Alexa Fluor probes (both at 1:1,000).

### LysoTracker staining

For LysoTracker staining, live (unfixed) tissues were put in 1:200 LysoTracker Red DND-99 (Molecular Probes) in PBS for 5 min, followed by three quick washes in PBS, mounted in Glycerol or Vectashield and immediately visualized under confocal microscope.

### Anti-DNP staining

For anti-DNP staining, brains were dissected in cold PBS, incubated for 30 min at 37 °C in 10 mM DAMP (*N*-(3-((2,4-dinitrophenyl) amino)propyl)-*N*-(3-aminopropyl) methylamine, dihydrochloride) (Life Technologies), and washed three times in PBS, before fixing. Rabbit anti-DNP-BSA (ICN ImmunoBiologicals) was used for DAMP detection.

### BrdU feeding

BrdU feeding (pulse chase) experiment was modified from von Trotha et al. [63]. Larvae were washed in PBS and starved for 3 h on filter paper (Whatman, Springfield Mill, Kent, UK) soaked with 5 % sucrose (Sigma-Aldrich), 1 mg/ml BrdU (GE Healthcare, Piscataway, NJ, USA) and 1 % red food color (Dr Oetker) (Supplementary Fig. S5). The food color was used as indication of animals that have digested BrdU.

### Transmission electron microscopy

Transmission electron microscopy was performed as described in [9]. Images were taken with JEOL EX 1200 II (Jeol Ltd.). TEM was equipped with Gatan Erlangshen ES5000W, model 782 CCD-camera (Gatan Inc.).

### Confocal microscopy and image analysis

Images were acquired by Leica TCS SP5 and processed with ImageJ, Photoshop and Bitplane Imaris suite. All confocal images presented are sections, apart from Fig. 2c (3-D reconstructions) and Supplementary Fig. S3b–e.

### Trauma induction

Trauma was induced as described in Leyssen et al. [34].

### Western blotting

Ten dissected brains of each genotype were processed according to manufacturer’s instructions (Amersham Biosciences). Animals were raised at 25 °C. The following antibodies were used: rabbit anti-twinfilin (1:2,000, [65]) and rabbit anti-DmMANF (1:1,000, [42]).

## Results

### Concurrent DmMANF knockdown and Dicer-2 overexpression in glia results in the appearance of an unusual cell type

*DmMANF* is the *Drosophila* ortholog of vertebrate *CDNF* and *MANF* genes. MANF and CDNF belong to a novel class of conserved neurotrophic factors that specifically protect and restore dopaminergic (DA) neurons in mammalian models [36, 64]. In *DmMANF* mutant larvae, the neurites of DA neurons are diminished [42]. *DmMANF* mutants are early larval lethal and this lethality can be rescued by *Drosophila* and human *MANF* genes, suggesting that *DmMANF* is a conserved secreted protein [42]. In addition to being secreted, MANF can bind intracellularly to the KDEL receptor in the endoplasmic reticulum (ER) [18]. Furthermore, several lines of evidence also suggest intracellular functions for MANF. Of these, its role as an ER stress-responsible protein has been demonstrated both in vitro [3] and in vivo [41].

**Fig. 1:**
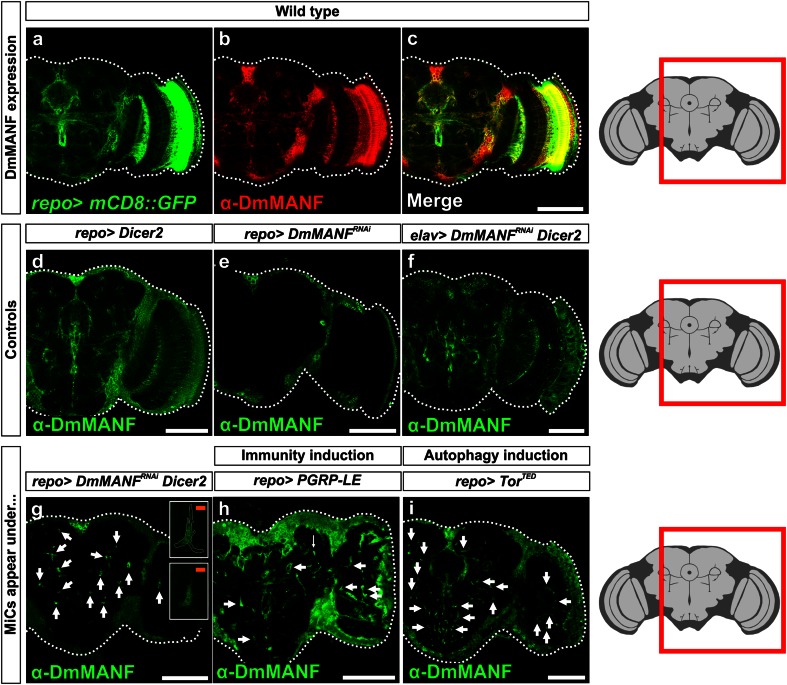
MiCs appear in pupal brain upon *DmMANF* knockdown and overexpression of *Dicer*-*2* in glia cells. **a**–**c** In wild-type animals, DmMANF is widely distributed and co-localizes with glial processes (*mCD8::GFP* membrane-tethered GFP). **d** *Dicer*-*2* overexpression does not have any obvious effect on DmMANF expression. DmMANF knockdown in glial cells (**e**) or concurrent *Dicer*-*2* overexpression and *DmMANF* knockdown in neurons (**f**) results in DmMANF downregulation. **g** *DmMANF* knockdown, along with *Dicer*-*2* overexpression in glial cells, results in the appearance of MiCs in late pupae (**g**, *arrows*), compared to controls (**d**–**f**). MiCs have elongated arms (**g**, *insets*; see also Fig. 2c). Concurrent *DmMANF* knockdown and *Dicer*-*2* overexpression is insufficient to remove all DmMANF expression. **h**, **i** MiCs also appear when either immunity (**h**, arrows; see also Fig. 9) or autophagy (**i**, *arrows*; see also Fig. 10) are triggered specifically in glial cells. *White scale bars* 100 μm, *orange scale bars* 10 μm. The *red square* on the brain sketch indicates the area that the confocal images correspond to. *Light grey* neuropil areas devoid of cell bodies; *dark grey areas* areas where cell bodies exist

Our previous study showed that during embryonic and larval stages, DmMANF is strongly expressed in cell body glia that are positive for the transcription factor Eagle. In addition, during embryogenesis weaker expression was seen in the longitudinal and channel glia, but no neuronal expression was detected [42]. Here we show that in the pupal and adult brains, DmMANF shows wider distribution. DmMANF co-localizes with *repo*>*UASmGFP*, indicating that DmMANF is located in glial processes (Fig. 1a–c). Interestingly, contrary to embryos [42], in the adult brain DA neuron cell somas are stained with DmMANF (Supplementary Fig. S1). DmMANF null mutants die early in development, as second instar larvae [42]. To explore the role of DmMANF in the pupal and adult brain, we used RNA interference (RNAi). The RNAi effect was also enhanced by concurrent *Dicer*-*2* overexpression (Fig. 1f, g), an approach that is commonly used in *Drosophila* [8]. We tested the RNAi construct by expressing it ubiquitously, and found that the *tub*-*Gal4*; *UAS*-*DmMANF* ^*RNAi*^ *UAS*-*Dicer*-*2* larvae died as young larvae phenocopying the *DmMANF* null mutant phenotype [42]. In addition, Western blot analysis demonstrated that the *DmMANF* ^*RNAi*^ construct specifically downregulates DmMANF (Supplementary Fig. S2).

When expressing *DmMANF* ^*RNAi*^ (combined with *Dicer*-*2* expression) in all glia using the pan-glial driver *repo*-*Gal4*, the mutant flies died in their pupal case. Only 3 % of the pupae eclosed to adults (*n* = 60). Staining their brains with anti-DmMANF revealed a dramatic phenotype in the late pupae: the appearance of numerous cell bodies in the brain neuropil, which is an area of the brain that neither neuronal nor glial cell bodies are known to populate. This phenotype that to the best of our knowledge has not been previously described in any context was detectable because these cells were MANF positive (Fig. 1g; Supplementary Movie S1). The phenotype was fully penetrant since all pupae examined manifested this phenotype [*n* > 300 (for details about all genetic crosses, see Supplementary Table S1)]. Based on this initial observation we named these cells MiCs (MANF Immunoreactive Cells). We found very intriguing that although we knocked down DmMANF in all glia, a cell type (MiC) that is positive for DmMANF appears. Therefore, we examined if they would also appear in any other conditions than DmMANF knockdown. Strikingly, from a panel of 116 different manipulations (Supplementary Table S1), we were able to recapitulate the same phenotype in two other instances: by inducing immunity in glia cells (Fig. 1h) and by inducing autophagy in glia cells (Fig. 1i) (see later for details).

**Fig. 2:**
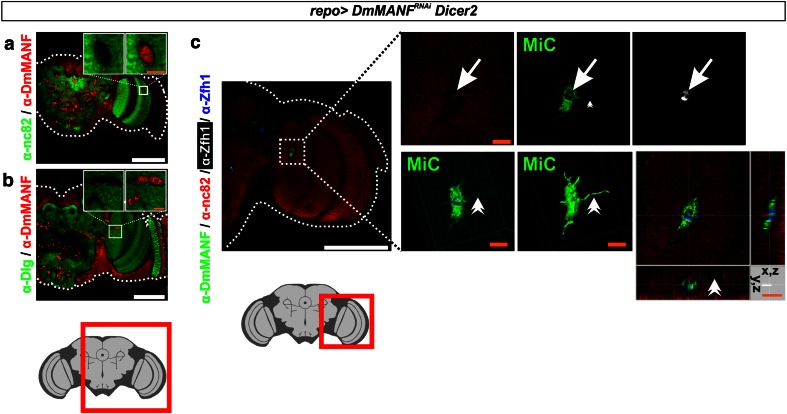
MiCs are found inside the neuropil. MiCs appear in sites where cell bodies are not expected, and they do not co-localize with the synaptic markers nc82 (**a**) and Dlg (**b**). **c** Orthogonal and 3-D views from the lobula area of the optic lobe show that MiCs are found inside the neuropil and they have elongated processes. *Arrows* indicate the relevant position of the nucleus (as indicated by the transcription factor Zfh1; MiCs are Zfh1 positive, see later for details). DmMANF is cytoplasmic and does not co-localize with the neuropil marker nc82. *Double arrowheads* indicate a single elongated process in 3-D and orthogonal views. *White scale bars* 100 μm, *orange scale bars* 10 μm. The *red square* on the brain sketch indicates the area that the confocal images correspond to. *Light grey* neuropil areas devoid of cell bodies; *dark grey areas* areas where cell bodies exist

MiCs were located in the neuropils, which are areas of the brain that are synapse-dense and filled with axons, dendrites and glial processes. Accordingly, neuropils are known to be devoid of all neuronal and glial cell bodies. Therefore the appearance of MiCs in these areas suggests that they are migratory. This was further supported by their elongated arms and cellular protrusions (Figs. 1g, inset, 2c). MiCs were also detected in great numbers in the ventral nerve cord neuropils of late pupae (Supplementary Fig. S3a), but not in non-neuronal tissues such as muscles (>2 h old male animals), testes or ovaries (>3 day old animals) (Supplementary Fig. S3c–e). Staining with the synaptic markers nc82 and Dlg revealed no staining at the sites where MiCs were found, indicating that they occupy a distinct area in neuropils (Fig. 2a, b) and that MiCs are found inside the neuropil (Fig. 2c).

Interestingly, MiCs never appeared when expressing independently either *UAS*-*Dicer*-*2* (Fig. 1d), or *UAS*-*DmMANF* ^*RNAi*^ (Fig. 1e), or combination of *UAS*-*Dicer*-*2* with other RNAi constructs in glia or in neurons (Supplementary Table S1). These results show that MiC appearance is not due to upregulation of *UAS*-*Dicer*-*2* expression (Fig. 1d). On the other hand, the reason for the absence of MiCs when only knocking down *DmMANF* (Fig. 1e) remains unclear. This result can be either due to the lower efficiency of *DmMANF* knockdown alone, or alternatively the synergistic effect of *Dicer*-*2* upregulation and *DmMANF* downregulation specifically in glia cells. Importantly, MiCs were not detected when knocking down *DmMANF* with pan-neuronal driver *elav*-*Gal4* either alone or in conjunction with *UAS*-*Dicer*-*2* (Fig. 1f and Supplementary Table S1).

### The JAK/STAT pathway is activated in MiCs

**Fig. 3:**
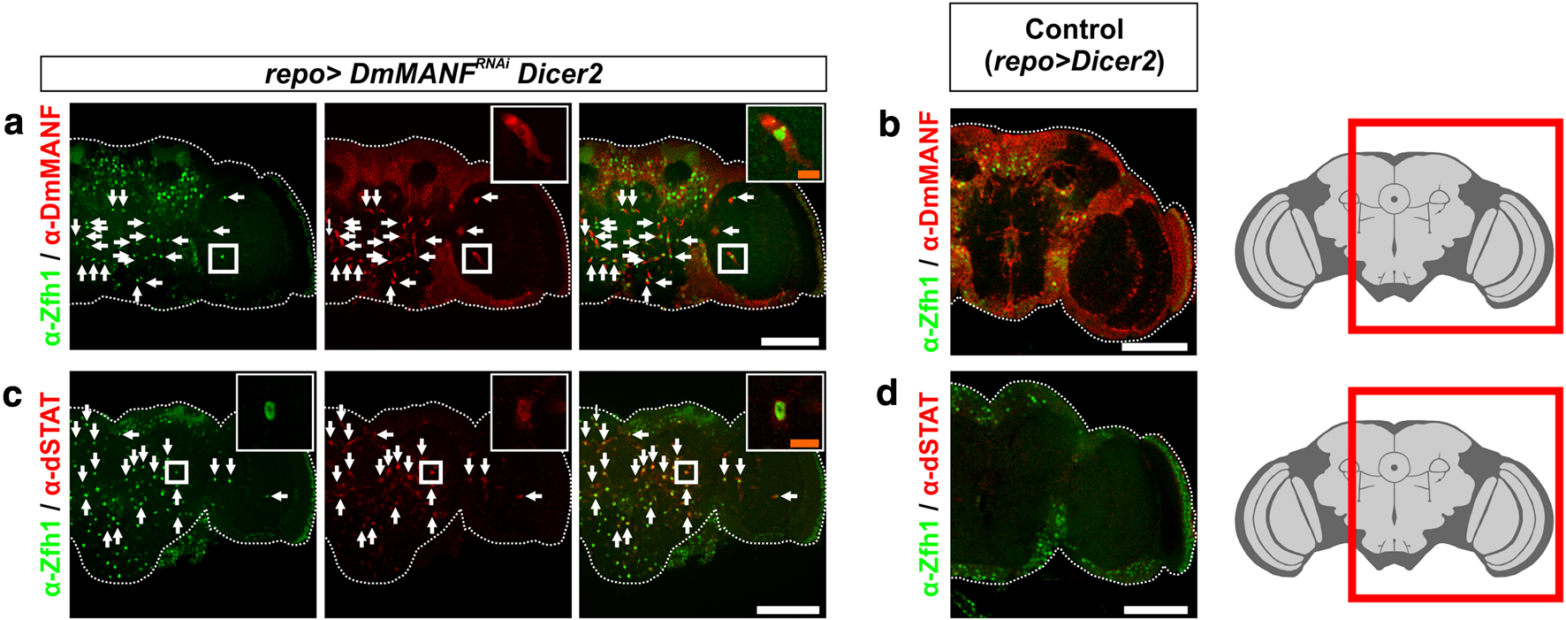
MiCs co-localize with the transcription factors Zfh1 and dSTAT. **a** MiCs express the transcription factor Zfh1, which is also used as a nuclear marker. **c** MiCs also express the transcription factor dSTAT. Importantly, dSTAT is nuclearly localized (*inset*), indicating it acts as an active transcription factor. **b**, **d** Control (*repo*>*UAS Dicer*-*2*) brains do not have MiCs, while Zfh1 and dSTAT expression is restricted at the periphery of the brain. *White scale bars* 100 μm. The *red square* on the brain sketch indicates the area that the confocal images correspond to. *Light grey* neuropil areas devoid of cell bodies; *dark grey areas* areas where cell bodies exist

The morphology of MiCs and their appearance in neuropil areas that are free of cell bodies suggest that they are motile. dSTAT activity is known to specify and maintain cell motility in various models of cell migration in *Drosophila*, including border cell migration [52], germ cell migration [68] and migration of embryonic tracheal cells [35]. We found that dSTAT is expressed in all MiCs (Fig. 3c) and more importantly it is accumulated in the nuclei (Fig. 3c, inset), which is a hallmark of JAK/STAT pathway activation [2]. Furthermore, all MiCs expressed the transcription factor Zfh1 (ZEB-1 homolog in vertebrates) (Fig. 3a), a known target of the activated JAK/STAT pathway [32], therefore confirming that the JAK/STAT pathway is activated in MiCs.

### MiCs are immune active cells

The JAK/STAT pathway has an evolutionarily conserved role in immune response and both dSTAT and Zfh1 have an established role in *Drosophila* innate immunity [1, 15, 25]. Innate immunity in flies is accomplished through two NF-*κ*B signaling pathways, the Toll and the Imd, which are similar to the mammalian Toll-like receptor (TLR) and Tumor Necrosis Factor (TNF) receptor pathways, respectively [21]. The Toll pathway is triggered by Gram-positive bacteria or fungi and can be activated by the ectopic expression of the Toll receptor. Activation of the Toll pathway results in nuclear translocation of the NF-κB factors DIF and dorsal which will trigger the expression of antibacterial response genes. The Imd pathway is activated by diaminopimelic acid containing peptidoglycans (PGN), commonly found at the cell walls of Gram-negative bacteria. These molecules bind to two peptidoglycan recognition proteins, PGRP-LC and PGRP-LE, which lead to nuclear translocation of the NF-κB factor Relish [21]. Translocation of Relish to the nucleus activates the transcription of antibacterial response genes, making it the key activator of the Imd pathway [11, 21]. Similarly to the Toll pathway, the Imd pathway can be activated by ectopic expression of either PGRP-LC or PGRP-LE [58].

**Fig. 4:**
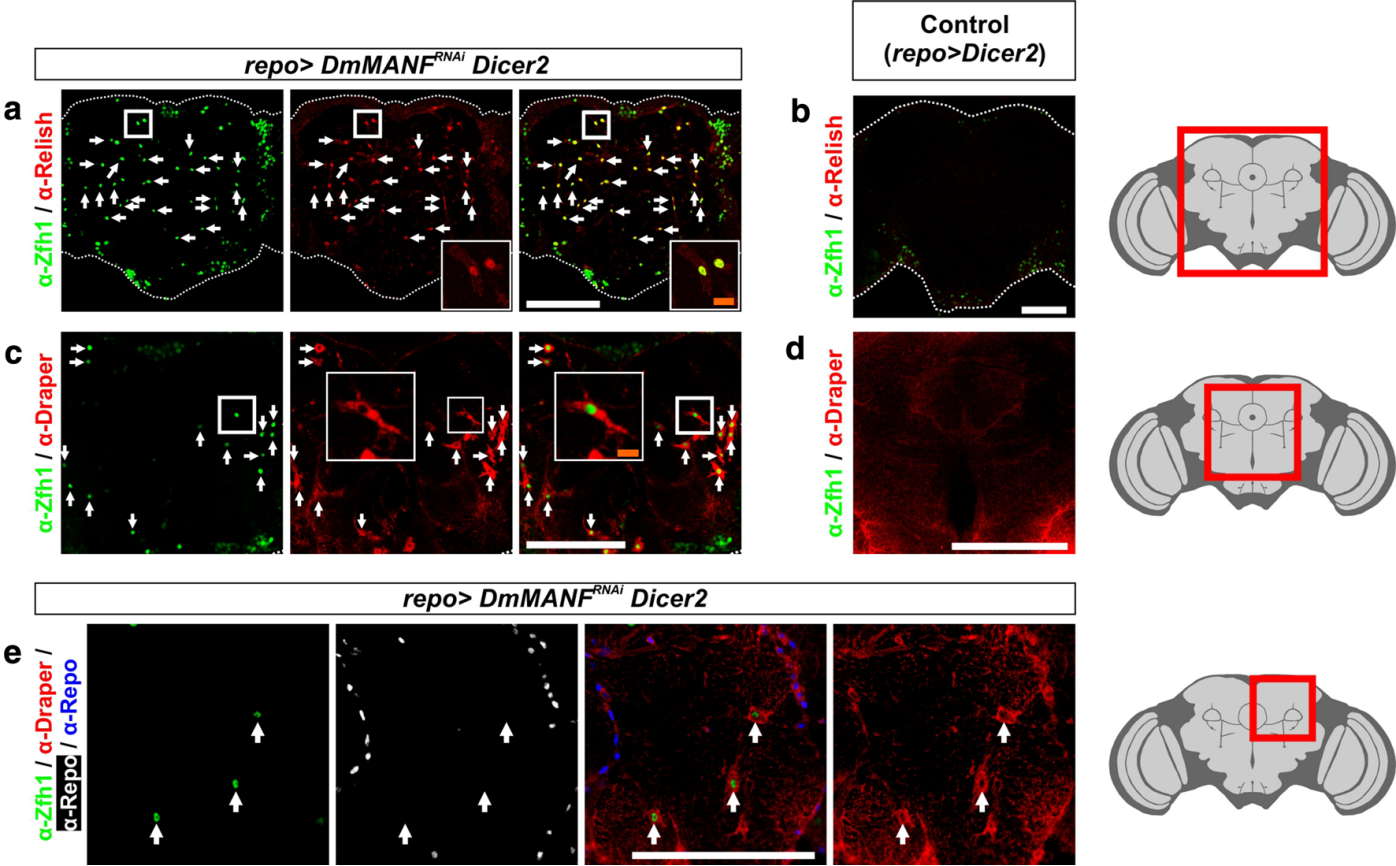
MiCs express the NF-κB factor Relish and the engulfment receptor Draper. **a** The downstream target of the Imd pathway, the NF-κB factor Relish, is nuclearly localized in MiCs. **c** The engulfment receptor Draper is also expressed in MiCs. **b**, **d** Control stainings. **e** Staining that differentiates Draper expression in MiCs (*arrows*) and Draper expression in glia. *White scale bars* 100 μm. The *red square* on the brain sketch indicates the area that the confocal images correspond to. *Light grey* neuropil areas devoid of cell bodies; *dark grey areas* areas where cell bodies exist

We found that MiCs express the key activator of the Imd pathway, the NF-κB factor Relish (Fig. 4a). Importantly, Relish is accumulated in the nucleus of MiCs, result that indicates that MiCs are immune active cells (Fig. 4a, inset). Notably, upon immune challenge, Relish is cleaved, the N-terminal fragment is translocated to the nucleus and the C-terminal fragment remains in the cytoplasm [57]; cleavage per se however, is not sufficient for Relish translocation [25]. Curiously, in MiCs we detected nuclear localization using antibodies recognizing both fragments (Supplementary Fig. S4). However, nuclear localization of the C-terminal fragment of Relish has recently been reported [59], result that is in accordance with our observation.

### MiCs express the phagocytosis marker Draper and are rich in lysosomes

The unexpected appearance and the morphology of MiCs resemble infiltrating macrophages/hemocytes or mammalian microglia which in turn suggest that they have phagocytic properties. *draper* is the *Drosophila* homolog of the *C.* *elegans* engulfment gene *ced*-*1* (also homolog to Jedi-1 and MEGF10 in mice) and is a key phagocytic receptor involved in all phagocytic functions of *Drosophila* glia [4, 16, 28, 37–39, 60]. We found that all MiCs express Draper (Fig. 4c, e), therefore they are potentially phagocytic.

**Fig. 5:**
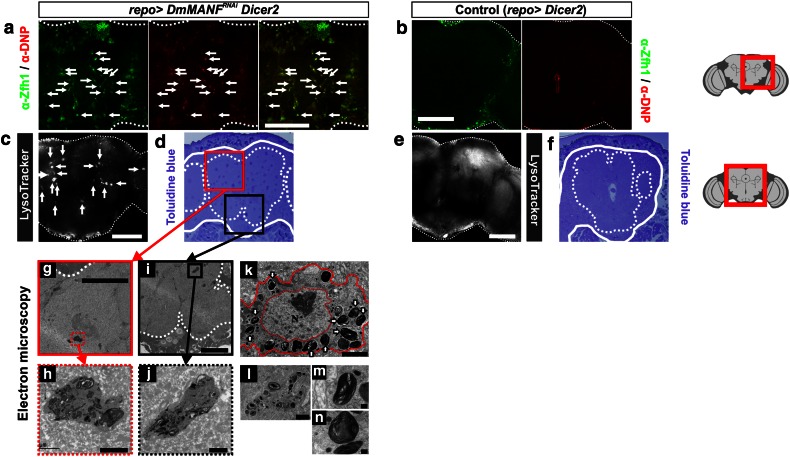
MiC are highly acidic, contain multiple lysosomes with abnormal structures. **a** MiCs take up DAMP (which is recognized by anti-DNP), indicating that MiCs are cells of high acidity. Lysosomes are organelles with highly acidic content. **b** Control brains do not take up DAMP. **c**, **e** *repo*>*DmMANF* ^*RNAi*^ *Dicer*-*2* brains are positive for Lysotracker (**c**), compared to control brains (**e**, *repo*>*Dicer*-*2*). In semi-thin sections, toluidine blue, which detects sulfate group elements, stains MiCs intensively (**d**), compared to control (**f**). Brain is surrounded by *closed line*, while neuropils are surrounded by *dotted lines*. Electron microscopy ultrathin sections show the existence of MiCs in neuropil areas (**g**, **i**). MiCs have a very high lysosomal content (**h**, **j**, **k**, **l**). Ultrastructure of MiCs reveals a cell whose nucleus is intact and has a high concentration of membranous structures (large lysosomes) (**k**) arranged in whorled arrays (**m**, **n**). *White scale bars* 100 μm, *black scale bars* 50 μm (**g**, **i**), 2 μm (**h**, **j**–**l**) and 0.2 μm (**m**, **n**); *N* nucleus, *arrows* lysosomes, *V* vacuoles. The *red square* on the brain sketch indicates the area that the confocal images correspond to. *Light grey* neuropil areas devoid of cell bodies; *dark grey areas* areas where cell bodies exist

To obtain cues about the function of MiCs we examined their ultrastructure. Semi-thin sections stained with toluidine blue revealed sparsely located cells of high acidic content (Fig. 5d). Transmission electron microscopy (TEM) identified cells in the neuropil areas with dramatic morphology. Their nuclei were intact indicating that they were alive. Their cytoplasm was filled with large lysosomes (Fig. 5k), which contained transversely stacked membranes (Fig. 5h, j–n). To verify that these cells are actually MiCs, we showed that MiCs take up DAMP, a molecule that is used to detect acidic organelles such as lysosomes (Fig. 5a). Such cells did not ever appear in the TEM samples of the control pupal brain (Fig. 5b, e–f).

### MiCs do not express the glial marker Repo, or the neuronal marker Elav

**Fig. 6:**
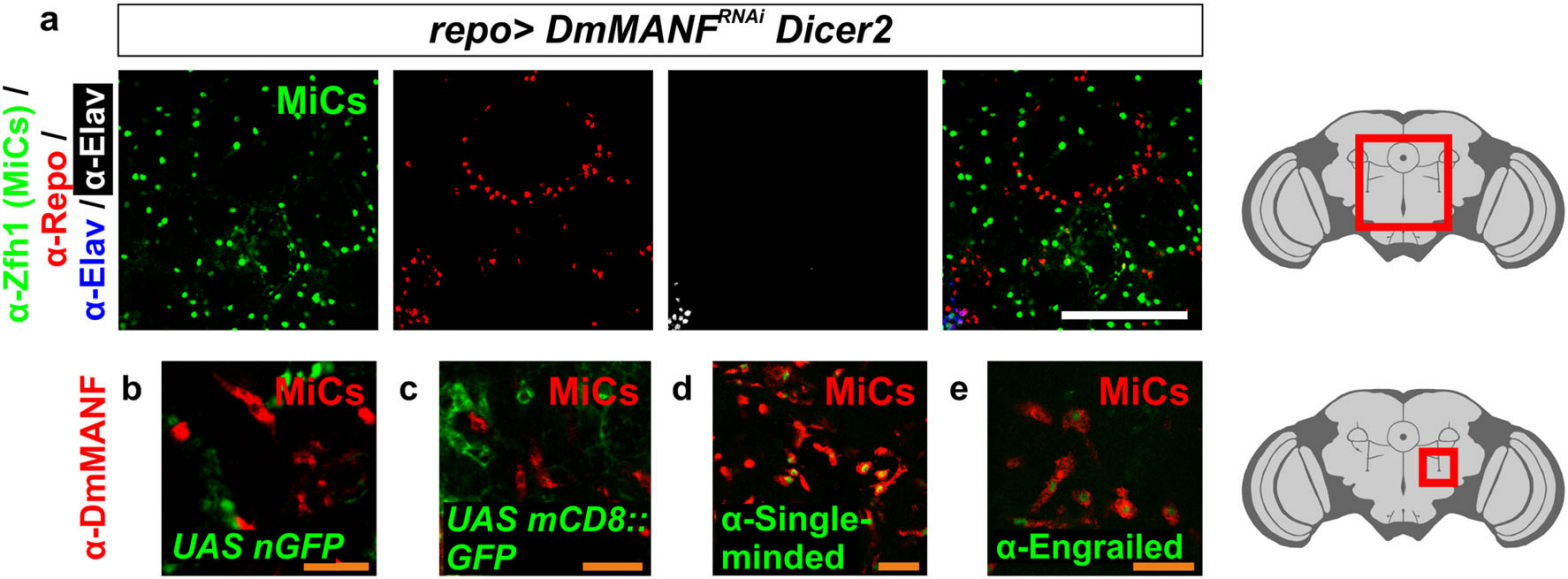
MiCs do not express glial and neuronal markers. **a** MiCs do not express Repo or Elav (MiCs are positive for Zfh1). **b**, **c** They also do not co-localize with Repo-positive cells when following Repo expression using nuclear GFP (nGFP) and membrane-tethered GFP (mCD8::GFP). **d** MiCs express the midline glial marker single-minded. **e** MiCs also express the transcription factor Engrailed. *White scale bars* 100 μm, *orange scale bars* 25 μm. *Red square* on the brain sketch indicates the area that the confocal images correspond to. *Light grey* neuropil areas devoid of cell bodies, *dark grey areas* areas where cell bodies exist

The *Drosophila* adult and late pupal brain is composed of neurons and glia. Neurons are detected by the pan-neuronal marker Elav and glia by the pan-glial marker Repo. Surprisingly, MiCs did not express either Elav or Repo (Fig. 6a). This makes MiCs the only known cell type in the CNS at this developmental stage that is not positive for either of these common neuronal or glial markers. Next, we followed the Repo expression with GFP (Fig. 6b, c). MiCs were not positive for GFP, indicating that at no developmental stage MiCs were expressing Repo. However, this experiment does not unambiguously rule out that MiCs could have been Repo positive earlier during development, as the GFP half-life under these conditions are unknown.

In addition to Repo-positive glia, flies have a small subset of glia called midline glia, which originate from mesoectoderm, but not from neuroectoderm where all other glial types in *Drosophila* originate. In wild-type flies, midline glia are eliminated by apoptosis during late embryogenesis and metamorphosis [48] and they do not exist in late pupae or adult flies. Midline glia do not express Repo at any developmental stage, but instead express the midline glia specific transcription factor Single-minded (Fig. 6d) [7]. Therefore, one possibility is that MiCs are descendants of midline glia which do not undergo apoptosis, but instead transdifferentiate into MiCs. In contrast with the hypothesis that MiCs derive from midline glia, we found that MiCs express Engrailed (Fig. 6e), a transcription factor that is known not to be expressed in midline glia [22], while they do not express Slit (data not shown), a second midline glial marker [47].

### MiCs appear during early-mid pupation and do not prevail after eclosion

**Fig. 7:**
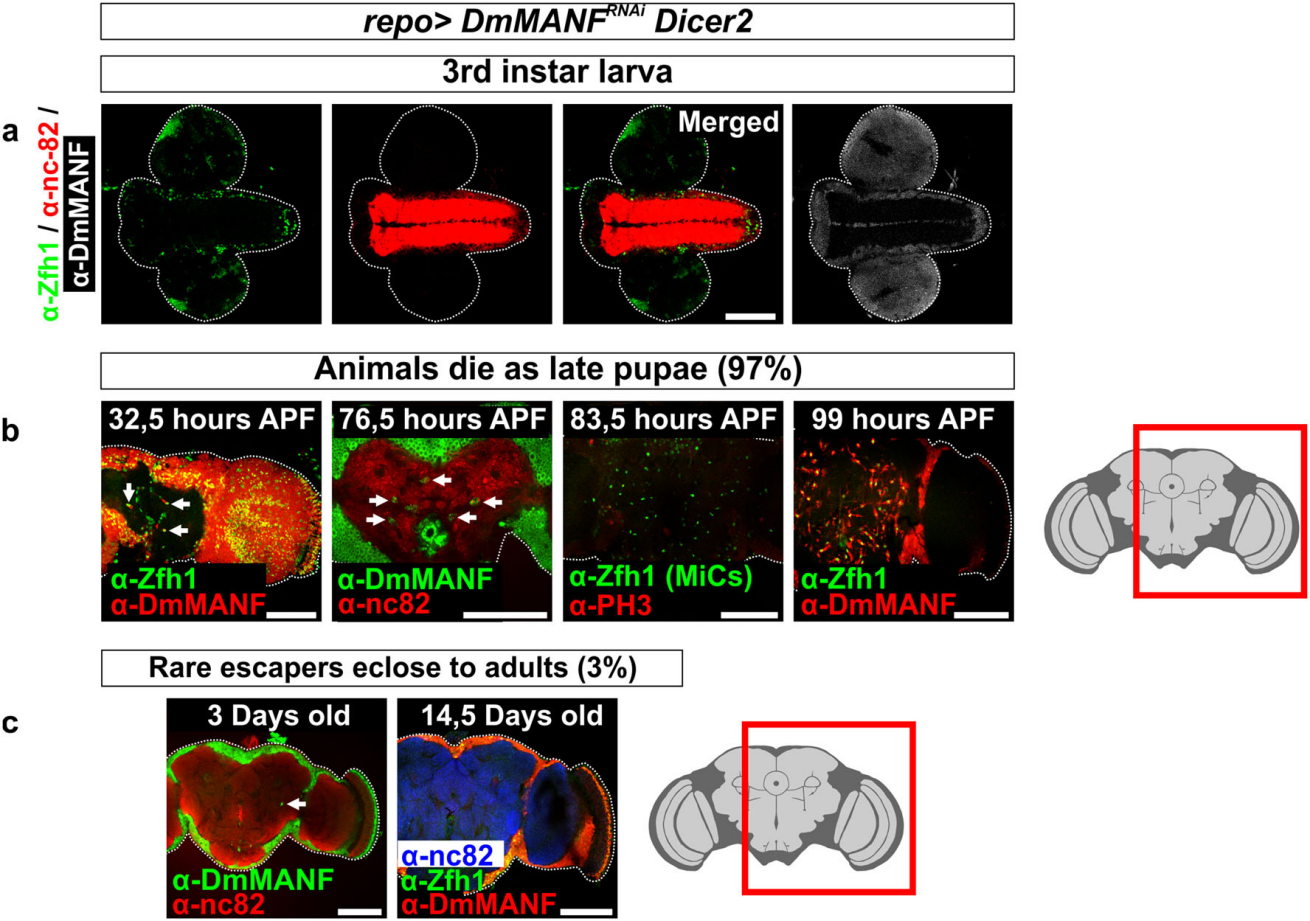
MiCs appear transiently during metamorphosis. **a** MiCs do not appear in the neuropil areas of third instar larva *repo*>*UAS*-*DmMANF* ^*RNAi*^ *UAS*-*Dicer*-*2* animals. **b** MiCs are apparent in the neuropil areas at 32.5 h APF (*arrows*), whereas their number increases greatly around 80 APF to reach their maximum at late pupation (99 h APF). During the time when MiCs increase in number, they do not co-localize with phospho-histone 3 (PH3) (83.5 h APF). **c** Rare adult escapers have virtually no MiCs in their neuropils. *White scale bars* 100 μm. *Red square* on the brain sketch indicates the area that the confocal images correspond to. *Light grey* neuropil areas devoid of cell bodies, *dark grey areas* areas where cell bodies exist

We followed the appearance of MiCs in larval and pupal brain at regular intervals. We never observed cells inside the neuropils of larval brains (Fig. 7a). The first time point we could observe Manf+/Zfh1+ cells inside the neuropil was at 32.5 h *after puparium formation* (APF) (Fig. 7b, 32.5 h APF). We also observed that the older the pupal brains were, the more MiCs they had. This observation is possibly related to the increase in the neuropil volume during the pupal brain development. Just before the expected eclosion time, MiCs were occupying large volume of the late pupal brain (Fig. 7b, 99 h APF); Supplementary Movie S1). The *repo*-*Gal4*; *UAS*-*DmMANF* ^*RNAi*^ *UAS*-*Dicer*-*2* late pupae were still alive, as upon opening of the pupal case, they were moving their proboscis and legs; however, they were unable to eclose from the pupal case.

**Fig. 8:**
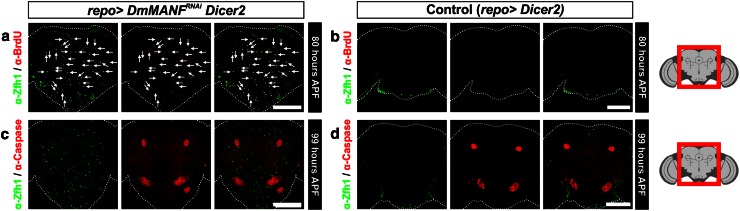
MiCs retain BrdU and are not caspase-3 positive. **a** Feeding experiments show that MiCs retain even at 80 h APF the BrdU incorporated at the larval stage (*arrows*). **c** MiCs do not express the apoptotic marker cleaved Caspase-3. *White scale bars* 100 μm. *Red square* on the brain sketch indicates the area that the confocal images correspond to. *Light grey* neuropil areas devoid of cell bodies; *dark grey areas* areas where cell bodies exist

Subsequently, we investigated whether MiCs are dividing during the pupal stage. MiCs were not positive for the mitotic marker phosphorylated histone 3 (PH3), indicating that MiCs do not proliferate during metamorphosis (Fig. 7b, 83.5 h APF). Next, we transiently fed third instar larvae before their wandering stage with BrdU (Supplementary Fig. S5) and we found that at 80 APF (more than 90 h after feeding) all MiCs were still positive for BrdU (Fig. 8a). This shows that progenitors of MiCs exist already at the larval stage, as MiCs took up BrdU. The amount of BrdU positive cells should halve in each cell division, and since all MiCs were positive for BrdU, this result suggests that during pupal stage, MiCs do not divide. Combining the PH3 staining and the BrdU incorporation results, it is unlikely that MiCs divide during pupal period.

Our results also show that Manf+/Zfh1+ cells inside the neuropil seldom exist in flies after eclosion. The rarely appearing adults (3 %) had very few or no Manf+/Zfh1+ cells inside their neuropils and they were never seen in flies more than 10 days old (Fig. 7c). Interestingly, staining with antibody against Caspase-3 showed that if MiCs disappear after eclosion, this is not due to caspase dependent apoptosis during late pupal stage (Fig. 8c).

### Induction of immunity in glia also results in the appearance of MiCs

**Fig. 9:**
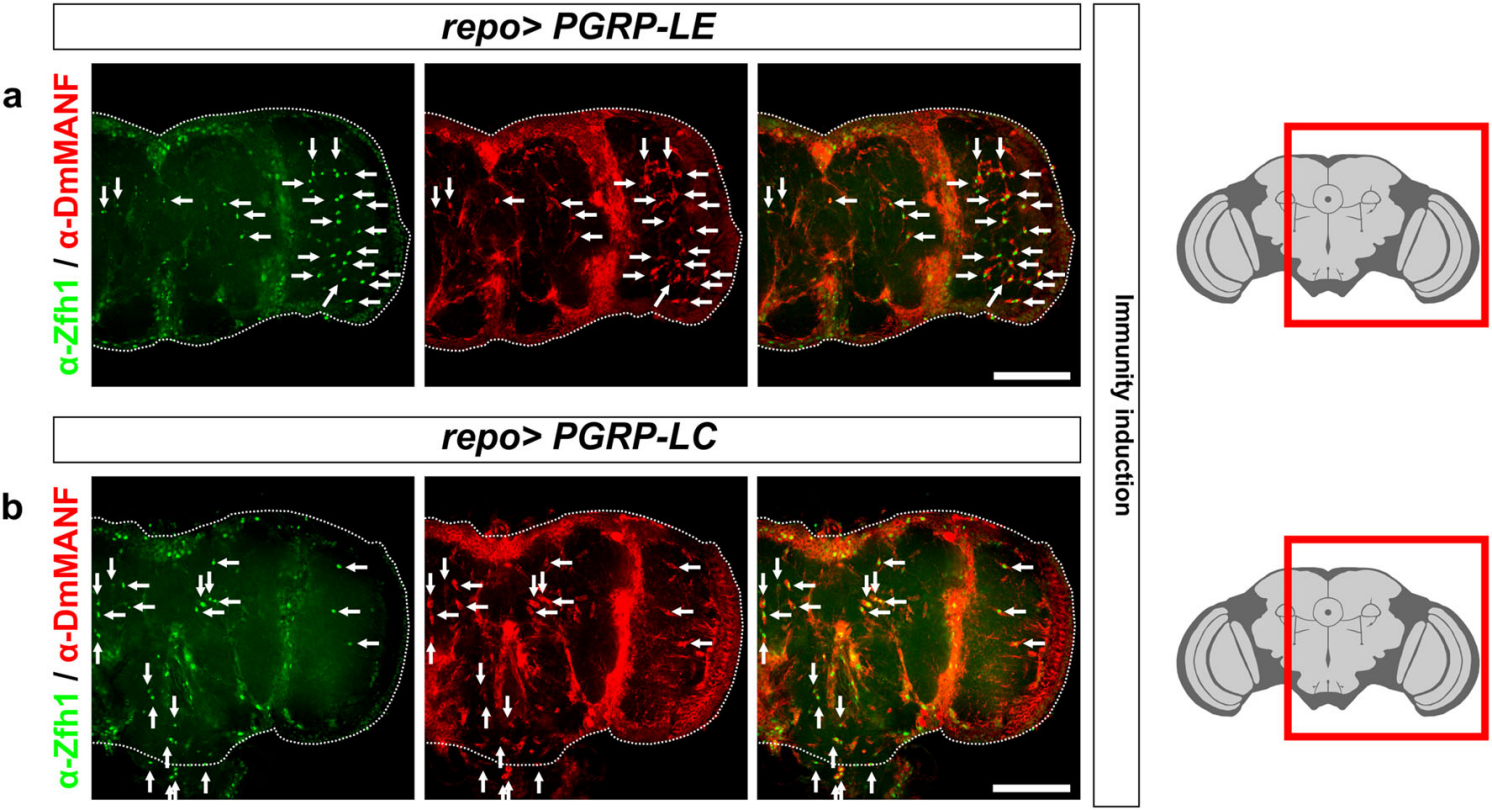
MiCs appear when the Imd pathway is activated in glia. MiCs appear when overexpressing in glia the PGRP-LE (**a**) and -LC (**b**) receptors, both of which are known to activate the Imd pathway. *White scale bars* 100 μm. *Red square* on the brain sketch indicates the area that the confocal images correspond to. *Light grey* neuropil areas devoid of cell bodies; *dark grey areas* areas where cell bodies exist

To explore whether the appearance of MiCs is only a *DmMANF*/*Dicer*-*2* related phenotype and to test if MiCs also appear in other contexts, we changed our focus to different genetic backgrounds. As shown above, in MiCs the JAK/STAT pathway is activated and Zfh1 is expressed, which are both implicated in immune response [1, 15, 25]. Therefore, we investigated if artificial induction of immune response in glia would induce the appearance of MiCs. We found that MiCs appeared when activating the Imd pathway in glia by ectopic expression of the PGN proteins *PGRP*-*LE* or *PGRP*-*LC* (Fig. 9a, b), but not when expressing the Toll receptor. Contrary to glia, the neuronal expression of any of these constructs did not produce MiCs (Supplementary Table S1).

### Induction of autophagy in glia also results in the appearance of MiCs

**Fig. 10:**
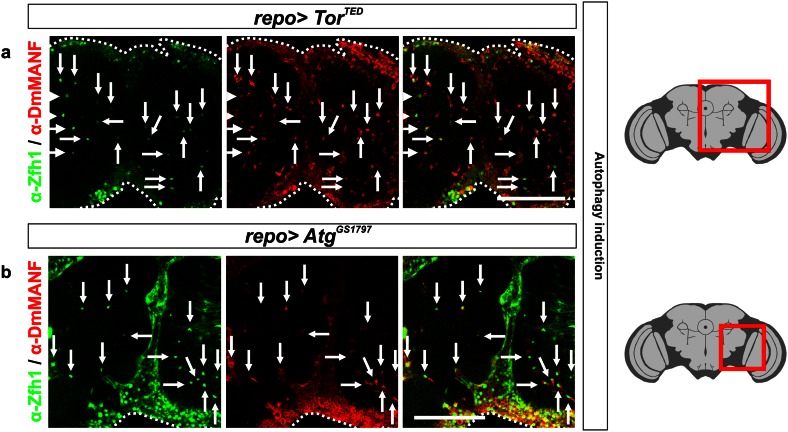
MiCs appear when autophagy is activated in glia. MiCs appear when autophagy is induced in glia cells, either by expressing a dominant-negative form of Tor (*Tor* ^*TED*^) (**a**) or by overexpressing *Atg1* (**b**). *White scale bars* 100 μm. *Red square* on the brain sketch indicates the area that the confocal images correspond to. *Light grey* neuropil areas devoid of cell bodies; *dark grey areas* areas where cell bodies exist

A second conserved defense mechanism between *Drosophila* and vertebrates to tackle pathogens is autophagy. Autophagy is a general term for pathways by which cytoplasmic material is delivered to lysosomes for degradation. Several observations implicate that MiCs were involved in autophagy: MiCs were rich in lysosomes and they expressed Draper, which has been shown to regulate autophagy in dying salivary glands [40]. In addition, PGRP-LE has been shown to trigger autophagic response [69]. To test if there is a link between MiCs and autophagy, we expressed *Atg1* [49] or the dominant-negative form of Target of rapamycin (*Tor* ^*TED*^) [50] in glia. Interestingly, both constructs recapitulated the phenotype when expressed using the *repo*-*Gal4* driver (Fig. 10a, b).

We took the advantage of the temperature sensitivity of the UAS/GAL4 system [10] and the higher permissive temperature of the *repo*>*TOR* ^*TED*^ animals (see Supplementary Table S1 for details) to determine the critical developmental period for the appearance of MiCs. We found this to be the first and second instar larval stage (Supplementary Fig. S6).

### MiCs induced by any of the three genetic manipulations express the same markers

**Fig. 11:**
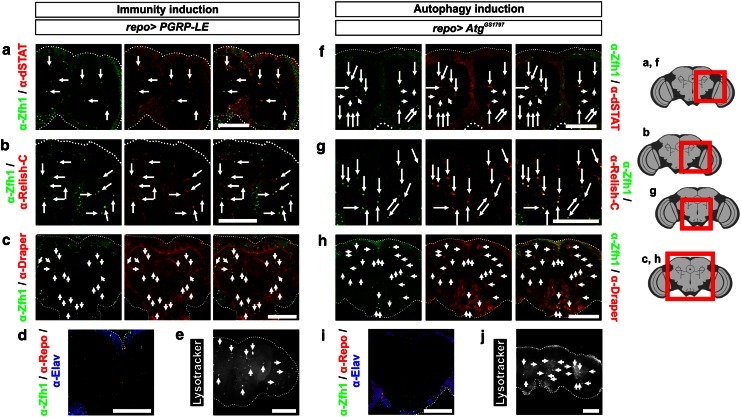
MiCs triggered by expression of *PGRP*-*LE* and *Atg1* ^*GS1797*^ also express dSTAT, Relish and Draper. As in the case of *repo*>*DmMANF* ^*RNAi*^ *Dicer*-*2*, MiCs triggered by *repo*>*PGRP*-*LE* also have nuclear accumulation of dSTAT (**a**), Relish (**b**) and they express Draper (**c**). **f**–**h** The same is true for MiCs triggered by *repo*>*Atg1* ^*GS1797*^. **d**, **i** In all cases that MiCs are induced, they do not co-localize either with Elav or Repo (for *repo*>*DmMANF* ^*RNAi*^ *Dicer*-*2*, see Fig. 6a). **e**, **j** MiCs induced under all genetic manipulations are positive for the lysosome marker LysoTracker (for *repo*>*Dicer*-*2*, see Fig. 5e; for *repo*>*DmMANF* ^*RNAi*^ *Dicer*-*2*, see Fig. 5c). *Scale bars* 100 μm. *Red square* on the brain sketch indicates the area that the confocal images correspond to. *Light grey* neuropil areas devoid of cell bodies; *dark grey areas* areas where cell bodies exist

To investigate if MiCs arising from either induction of immunity or induction of autophagy in glia are analogous to MiCs that appear by concurrent downregulation of *DmMANF* and overexpression of *Dicer*-2 in glia, we examined whether they expressed the same markers. In all cases MiCs are positive for all the markers tested, namely the neurotrophic factor DmMANF, Zfh1h and Draper (Figs. 3a, 4b, 11). Also in all situations dSTAT and Relish were accumulated in the nuclei of MiCs (Figs. 3c, 4a, 11). In addition, they did not express with either Repo or Elav (Figs. 6a, 11d, i). Furthermore, all challenged phenotypes were positive for Lysotracker (Figs. 5d, 11e, j). Accordingly, we conclude that MiCs arising under any of the three genetic manipulations are the same cell type.

### MiCs do not appear under other conditions

Of a total of 116 different manipulations, we observed MiCs only when inducing either immunity, or autophagy, or by concurrent downregulation of DmMANF and Dicer-2 overexpression specifically in glia. We never observed MiCs when inducing the same mechanisms in neurons, hemocytes, in subpopulations of glia using more specific drivers than *repo*-*Gal4*, or the early glial/hemocyte driver *gcm*-*Gal4* [62] (Supplementary Table S1). These results indicate that either the MiC phenotype can only be mediated by a global response in glia, or that the expression level of Gal4 in the sub-glial driver lines used is not strong enough. Furthermore, mRNA in situ hybridization analysis revealed that *gcm* mRNA is not expressed in *repo*-*Gal4*; *UAS*-*DmMANF* ^*RNAi*^ *UAS*-*Dicer*-*2* late pupal brains (data not shown).

We did not observe MiCs when performing a series of other manipulations in either glia or neurons (Supplementary Table S1). These manipulations include inhibition and activation of apoptosis by expressing the pro-apoptotic genes *rpr*, *hid* and *grim* or the anti-apoptotic genes p35 and DIAP1. We also did not observe MiCs when using the *swiss cheese* [27] and the *ATM* ^*8*^ [46] neurodegeneration models or using the alpha-synuclein model of *Drosophila* Parkinson’s disease [13]. Furthermore, MiCs were not seen when brain systemic stress was induced by heat shock in larvae or when brain trauma was inflicted in adult animals [34].

In addition, we investigated if MiCs appear when we used the hemocyte drivers hemese and hemolectin. We found cells within the pupal brain that express the hemocyte markers hemese [29] and hemolectin [19]. However, in contrast to MiCs both of these cell populations express Repo (Supplementary Fig. S7) indicating that they are glia rather than hemocytes and therefore making their lineage uncertain [33]. Interestingly, when we expressed either the *UAS*-*DmMANF* ^*RNAi*^ *UAS*-*Dicer*-*2* or the *UAS*-*PGRP*-*LE* or the *UAS*-*PGRP*-*LC* constructs under the drivers hemese and hemolectin, no MiCs appeared.

## Discussion

**Fig. 12:**
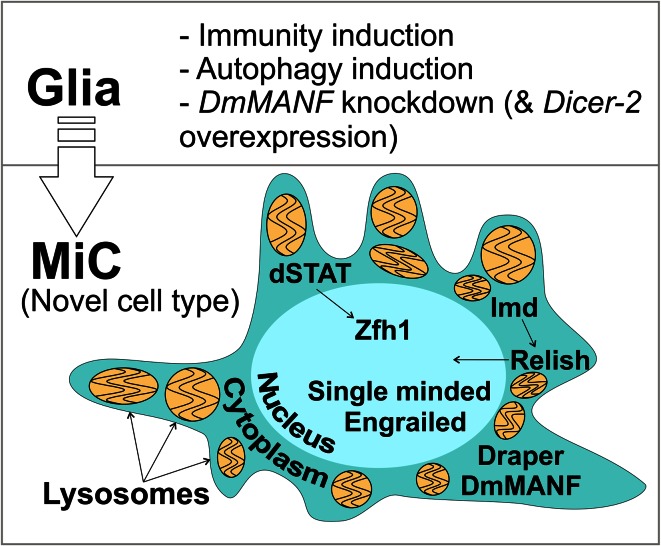
Summary of the main findings. In the pupal brain, when specifically in glial cells either (1) immunity or (2) autophagy or (3) concurrent *DmMANF* knockdown and *Dicer*-2 overexpression are induced, an unusual cell type appears. We call this type MiC. MiCs express the transcription factor Zfh1 and have nuclearly accumulated dSTAT and Relish. In addition, they are positive for the transcription factors Engrailed and Single-Minded, the conserved neurotrophic factor DmMANF and the engulfment receptor Draper. MiCs are loaded with lysosomes with multilamellar structures

Here we report the identification of an unusual cell type, that we call MiC, in the *Drosophila* brain (Fig. 12). The appearance of MiCs was induced by three mechanisms: the induction of either immunity, or autophagy, or when the conserved neurotrophic factor DmMANF was downregulated, specifically in glia cells. We conclude that in all three cases the cell type is the same because they are positive for the same markers, namely DmMANF, dSTAT, Zfh1, Relish and Draper, while they do not express Repo or Elav. MiCs were not observed when the same manipulations were done in neurons or in hemocytes or if they were limited only to subpopulations of glia. They were also not seen when using previously described *Drosophila* neurodegeneration models or various other manipulations (Supplementary Table S1).

### MiCs’ function

Our data suggest that MiCs produce an immune response. In MiCs, the JAK/STAT pathway is activated and the NF-κB factor Relish, which is the key activator of antibacterial peptide genes, is localized in the nuclei of MiCs. Consistent with this, it has recently been shown that the expression of constitutively active Relish in glia is sufficient to activate innate immune response and cause neurodegeneration in adult flies [6, 45].

In addition, we provide evidence that MiCs are potentially phagocytic. First, MiCs express the engulfment receptor Draper, a protein that is essential and required for engulfment in a number of studies [4, 16, 28, 37–40, 60]. Furthermore, MiCs have a very high lysosomal content, which suggests that they have a phagocytic function. On the other hand, we were not able to identify signs of endocytosis in MiCs, such as membrane internalizations or cellular debris. Consistent with our observations, recent data indicate that phagocytosis is not essential for microglia activation [23, 26, 44, 53] although they have phagocytic potential [44].

Our data also point towards that MiCs are motile cells. First, they have elongated arms typical for migrating cells. Second, they have a random distribution in the brain appearing in neuropil areas which are known to be devoid of all cell bodies. Finally, dSTAT, a transcription factor which is known to specify and maintain cell motility, is localized in the nuclei of MiCs [2, 35, 52, 68].

The functions described above, namely motility [23, 24, 66], production of pro-inflammatory mediators [23, 24, 66], expression of engulfment receptors [43, 66], being positive for neurotrophic factors and more specifically the neurotrophic factor MANF [23, 51] are all features of macrophages/hemocytes and mammalian microglia. In addition, their appearance only in the CNS and the ventral nerve cord as well as their mode of emergence under brain homeostasis disturbance, resembles activation of mammalian microglia.

There is some evidence for the existence of microglia-like cells in other invertebrates such as leeches and mollusks [23]. In cockroaches they have been reported to appear under in vitro conditions [54]. However, microglia have not been identified in *Drosophila*. Rather, in flies glia are competent to perform immune-like functions such as engulfment of neuronal corpses during development and adulthood.

### MiCs’ origin

In vertebrates, microglia have been studied for more than 100 years. However, until recently their origin has been under controversy (for review see [17]). Microglia, unlike glia and neurons, do not derive from the neuroectoderm. Instead they derive from macrophages produced by primitive hematopoiesis in the yolk sac [17, 24]. Similar to mammalian microglia, MiCs could also be of hematopoietic origin. In flies, macrophages/hemocytes, microglia or microglia-like populations have not been described in the CNS. MiCs did not appear when using hemocyte-specific Gal4 drivers either to knockdown *DmMANF* (and overexpress *Dicer*-*2*) or to induce immunity (Supplementary Table S1). Another possibility is that MiCs are circulating macrophages/hemocytes that infiltrate the brain upon genetically challenged conditions that may result in blood brain barrier disruption. Unfortunately, blood brain barrier disruption has not been studied during pupation and currently no experimental method exists for investigating blood brain barrier integrity. However, our in situ hybridization data show that *repo*-*Gal4*; *UAS*-*DmMANF* ^*RNAi*^ *UAS*-*Dicer*-*2* late pupal brains are not positive for the hemocyte marker *gcm* [62], therefore MiCs cannot be (at least typical) hemocytes.

Alternatively, MiCs may originate from midline glia. As MiCs, midline glia do not express Repo. They are of mesoectodermal origin and have a distinct lineage from all other glial cells. During normal development, midline glia are eliminated by apoptosis in two temporally distinct waves, which results in midline glia not existing during late pupation and in adulthood [48]. Interestingly, MiCs express the midline glia marker Single-minded. Therefore, it could be that MiCs indeed are midline glia that are not eliminated by apoptosis, but instead invade the neuropil areas. On the other hand, MiCs do not express the midline glia marker Slit, while they express Engrailed, a transcription factor that is not expressed in midline glia.

An exciting possibility is that MiCs may be either glia or neurons that under genetically challenged conditions transdifferentiate to MiCs and lose the expression of glial or neuronal markers. Very recently, a phagocytic cell type has been identified in *Drosophila* pupal brain [60]. These cells are glia, they express Draper and they are Lysotracker positive. However, in contrast to MiCs these cells appear in the wild-type brain and are Repo positive. In addition they are localized specifically at the periphery of the neuropil and extend only their processes inside the neuropil [60]. Finally, at the ultrastructural level (TEM) they do not show the characteristic MiC phenotype, namely multiple large lysosomes filled with transversely stacked membranes.

MiCs appear only transiently during metamorphosis, when a profound reorganization of the larval to adult CNS occurs. This cellular behavior may be vestigial from the evolution of Holometabola from hemimetabolous ancestors and it would be interesting to see if similar cells exist in normal conditions during brain development in species that undergo various forms of metamorphosis [30]. We propose that MiCs differentiate from an earlier established cell population and do not divide or divide at very low rate during metamorphosis. Two lines of evidence support this assumption: first, MiCs do not stain with the mitotic marker PH3 and second, in the pupal brain the BrdU fed during larval stage is retained.

## Conclusions

In summary, we show that by employing three different genetic mechanisms in vivo an unusual cell type appears in the *Drosophila* brain that we call MiC. MiCs express a unique set of molecular markers. These cells share many similarities with professional macrophages/hemocytes and vertebrate microglia. Macrophages/hemocytes, or microglia-like cells have not been previously identified in the *Drosophila* CNS. In addition, the pathways activated in MiCs, as well the molecular markers presented in this study, are evolutionarily well conserved from flies to humans, therefore making our results potentially relevant to higher organisms. Further investigations of MiCs’ origin, differentiation and stimuli that trigger them will help us to better understand how immunity is attained in the CNS.

## Electronic supplementary material

Below is the link to the electronic supplementary material.

Supplementary material 1 (XLSX 23 kb)

Supplementary material 2 (AVI 1775 kb)

Supplementary material 3 (PDF 1702 kb)

## Abbreviations

APF: After puparium formation
CNS: Central nervous systems
DA: Dopaminergic
ER: Endoplasmic reticulum
MiC: Manf immunoreactive cell
PH3: Phosphorylated histone 3
RNAi: RNA interference
TEM: Transmission electron microscopy
TLR: Toll-like receptor
TNF: Tumor necrosis factor
Tor: Target of rapamycin
