# Supplementary material for: MANF silencing, immunity induction or autophagy trigger an unusual cell type in metamorphosing Drosophila brain
Source: Cell Mol Life Sci. 2014 Dec 16;72(10):1989–2004. doi: 10.1007/s00018-014-1789-7 (PMC4412683; doi:10.1007/s00018-014-1789-7)
Supplement: Supplementary file 3 — Supplementary material 3 (PDF 1702 kb) [file 18_2014_1789_MOESM3_ESM.pdf]

Supplementary Figures

MANF silencing, immunity induction or autophagy trigger an unusual cell type in metamorphosing *Drosophila* brain

Vassilis Stratoulias[1], **Tapio I. Heino**[1*]

[1]Department of Biosciences, University of Helsinki, FI-00014 Helsinki, Finland.

*** Corresponding author:** T.I. Heino, Department of Biosciences, University of Helsinki, P.O. Box 56 (Viikinkaari 5), FI- 00014 Helsinki, Finland. E-mail: tapio.heino@helsinki.fi

Supplemental Inventory

Supplementary Fig. S1.

DmMANF also co-localizes with dopaminergic cell bodies.

Supplementary Fig. S2.

DmMANF knockdown in glia cells reduces DmMANF protein levels in brain lysates.

Supplementary Fig. S3.

MiCs in the ventral nerve cord and DmMANF localization in non-neuronal tissues.

Supplementary Fig. S4.

In MiCs, Relish is accumulated in the nucleus while also localized in the cytoplasm.

Supplementary Fig. S5.

BrdU feeding experiment set up.

Supplementary Fig. S6.

First and second instars are the critical developmental time for MiC induction.

Supplementary Fig. S7.

Hemocyte markers Hemese and Hemolectin are expressed in the pupal CNS in wild type animals.

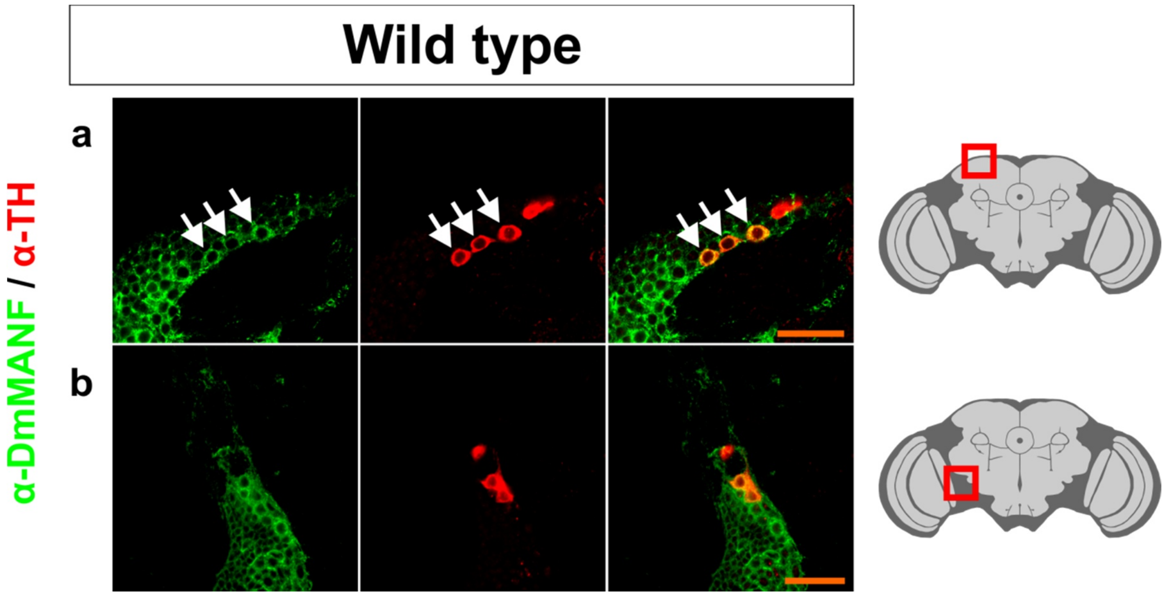

Supplementary Fig. S1.

DmMANF also co-localizes with dopaminergic cell bodies.

DmMANF, apart from glial processes, it also co-localizes with dopaminergic cell bodies. See co-localization in the PAL cluster (a) and in the PPL2 cluster (b). Orange scale bars, 25 µm. Note: the red square on the brain sketch indicates the area that the confocal images correspond to. Light grey: Neuropil areas devoid of cell bodies; Dark grey areas: Areas where cell bodies exist.

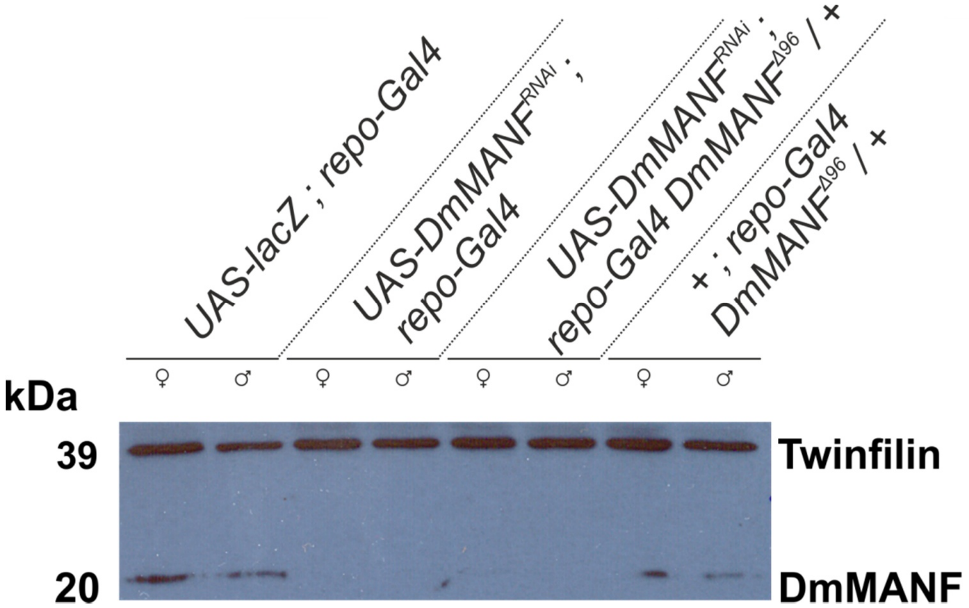

Supplementary Fig. S2.

DmMANF knockdown in glia cells reduces DmMANF protein levels in brain lysates.

The *DmMANF*^{RNAi} construct specifically targets for DmMANF mRNA degradation. Twinfilin serves as loading control. *DmMANF*$^{\Delta 96}$ is a null *DmMANF* allele [42].

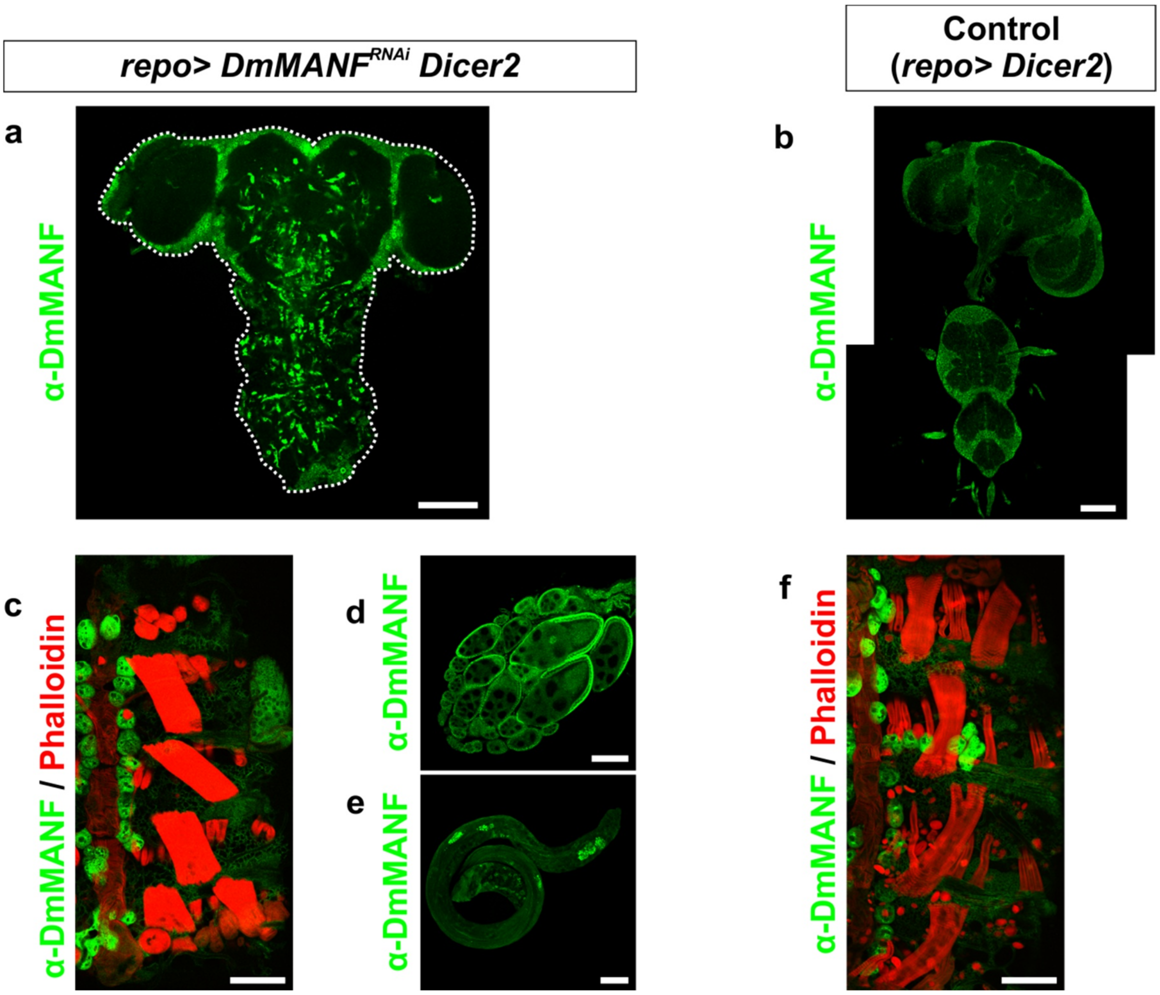

Supplementary Fig. S3.

MiCs in the ventral nerve cord and DmMANF localization in non-neuronal tissues.

(**a-b**) MiCs are found both in the brain and in the ventral nerve cord of pupae (a), in contrast to the control brain and ventral nerve cord that MiCs do not exist in the neuropil areas (b). (**c, f**) Pericardial cells (abdominal nephrocytes) are positive for DmMANF. (**d-e**) DmMANF is also expressed in the follicle cells of the adult ovary (d), and in investment cone bundles of adult testis (e). Although MiCs are found in the brain and ventral nerve cord of late pupae in large numbers (a), we could not identify them in very young muscles (< 2 hours old males in c), and in young ovaries and testes (< 3 days old animals in d-e). White scale bars, 100 μm.

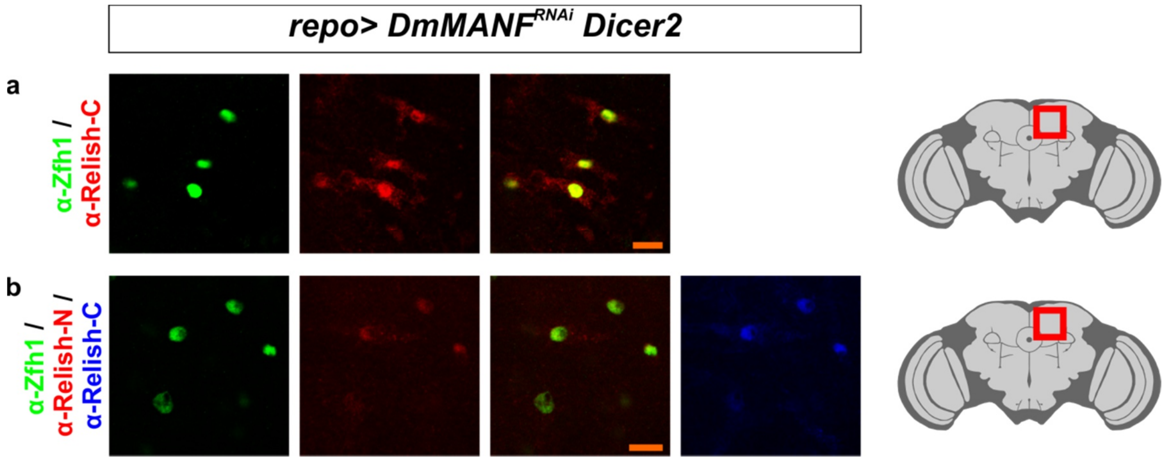

Supplementary Fig. S4.

In MiCs, Relish is accumulated in the nucleus while also localized in the cytoplasm.

(**a-b**) Antibodies raised against the C- and the N- terminus of Relish, both localize in the cytoplasm and the nucleus. Zfh-1 is a transcription factor that is expressed in MiCs and it has been used as a nuclear marker throughout the article. Orange scale bars, 10 μm. Note: the red square on the brain sketch indicates the area that the confocal images correspond to. Light grey: Neuropil areas devoid of cell bodies; Dark grey areas: Areas where cell bodies exist.

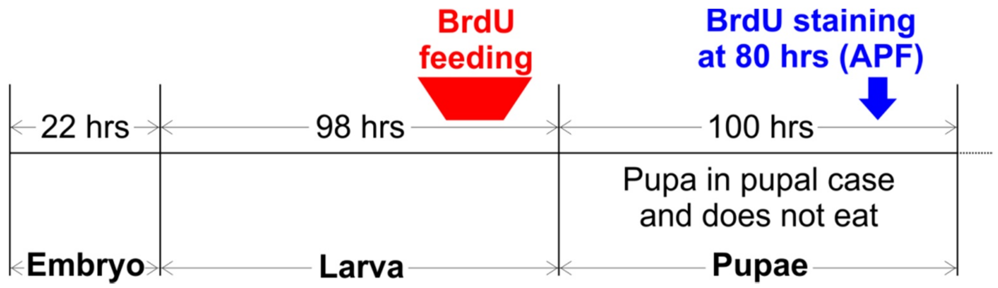

Supplementary Fig. S5.

BrdU feeding experiment set up.

Animals were fed transiently during larval stages with BrdU. Animals were stained at 80 hrs APF.

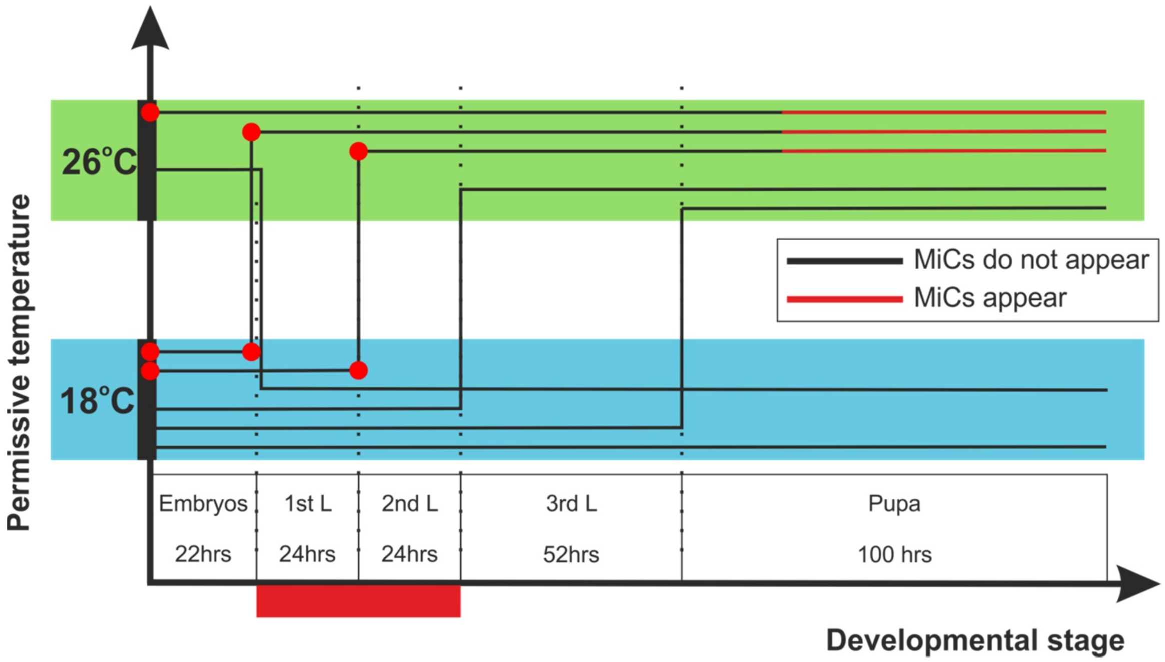

Supplementary Fig. S6.

First and second instars are the critical developmental time for MiC induction

At 18ºC the UAS-GAL4 system has minimal activity while its activity is significantly increased at 26ºC. *repo>Tor* ^{TED} were shifted between these two temperatures (y axis) at different developmental stages as indicated on the x axis (black lines). In the case that MiCs appeared in late pupal brains the line color changes into red. These experiments indicate that the developmental window between 1st instar and the end of 2nd instar larva is the critical time for induction of MiCs.

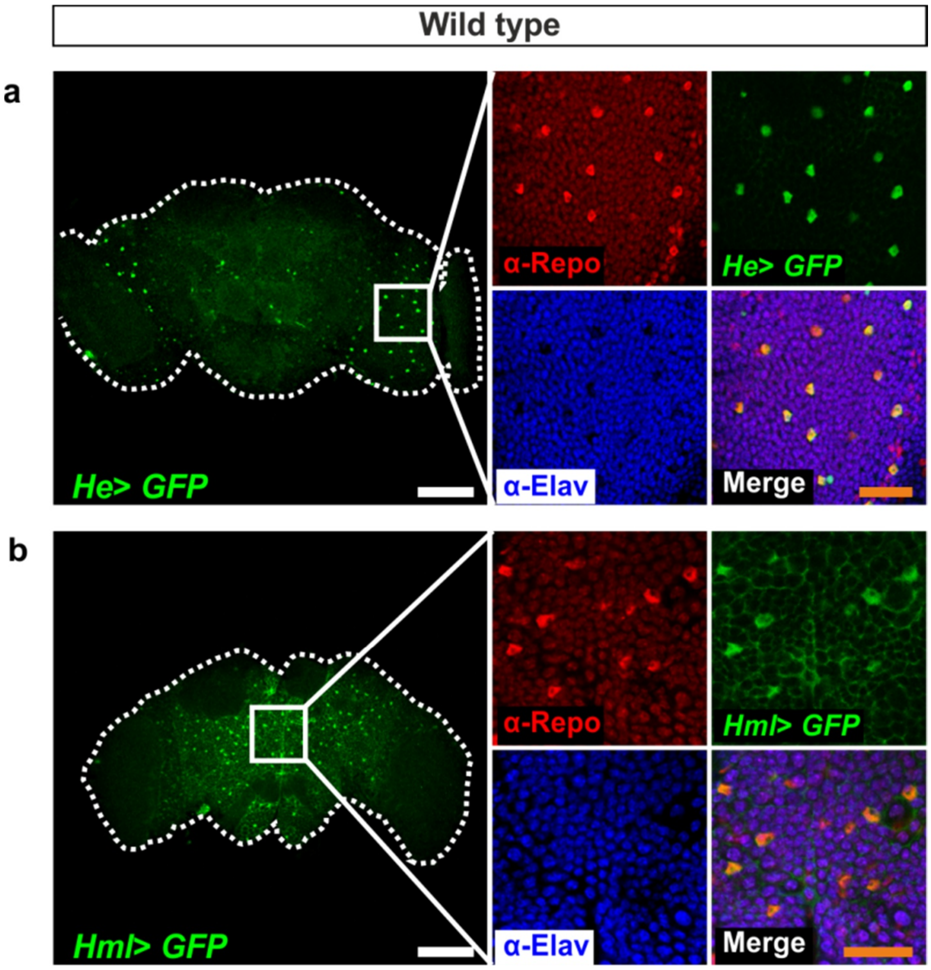

Supplementary Fig. S7.

Hemocyte markers Hemese and Hemolectin are expressed in the pupal CNS in wild type animals.

(**a-b**) Within the pupal and adult brain of wild type animals, cells that express the hemocyte markers Hemese (He) and Hemolectin (Hml) exist. They both co-localize with the glial marker Repo, but not with the neuronal marker Elav, result that makes their lineage uncertain. White scale bars, 100 µm; orange scale bars, 10 µm.
